# Left-Ventricular Energetics in Pulmonary Arterial Hypertension-Induced Right-Ventricular Hypertrophic Failure

**DOI:** 10.3389/fphys.2017.01115

**Published:** 2018-01-09

**Authors:** June-Chiew Han, Sarah-Jane Guild, Toan Pham, Linley Nisbet, Kenneth Tran, Andrew J. Taberner, Denis S. Loiselle

**Affiliations:** ^1^Auckland Bioengineering Institute, The University of Auckland, Auckland, New Zealand; ^2^Department of Physiology, The University of Auckland, Auckland, New Zealand; ^3^Department of Engineering Science, The University of Auckland, Auckland, New Zealand

**Keywords:** cardiac efficiency, ventricular trabeculae, working-heart, right heart failure, pressure overload

## Abstract

Pulmonary arterial hypertension (PAH) alters the geometries of both ventricles of the heart. While the right ventricle (RV) hypertrophies, the left ventricle (LV) atrophies. Multiple lines of clinical and experimental evidence lead us to hypothesize that the impaired stroke volume and systolic pressure of the LV are a direct consequence of the effect of pressure overload in the RV, and that atrophy in the LV plays only a minor role. In this study, we tested this hypothesis by examining the mechanoenergetic response of the atrophied LV to RV hypertrophy in rats treated with monocrotaline. Experiments were performed across multiple-scales: the whole-heart *in vivo* and *ex vivo*, and its trabeculae *in vitro*. Under the *in vivo* state where the RV was pressure-overloaded, we measured reduced systemic blood pressure and LV ventricular pressure. In contrast, under both *ex vivo* and *in vitro* conditions, where the effect of RV pressure overload was circumvented, we found that LV was capable of developing normal systolic pressure and stress. Nevertheless, LV atrophy played a minor role in that LV stroke volume remained lower, thereby contributing to lower LV mechanical work output. Concomitantly lower oxygen consumption and change of enthalpy were observed, and hence LV energy efficiency was unchanged. Our internally consistent findings between working-heart and trabecula experiments explain the rapid improvement of LV systolic function observed in patients with chronic pulmonary hypertension following surgical relief of RV pressure overload.

## Introduction

In pulmonary arterial hypertension (PAH), the increased pulmonary vascular resistance and pulmonary artery pressure impose pressure overload on the right ventricle (RV). The increased workload of the RV then initiates RV hypertrophy, and sustained hypertrophy subsequently leads to right-ventricular failure (RVF). PAH is not solely limited to dysfunction in the RV, however. In fact, the left ventricle (LV) suffers geometrical and functional alterations as well. There is a high degree of “ventricular interdependence” (Hsia and Haddad, 2012; Meyer, 2014) as the ventricles share the septum and compete for the limited and non-compliant pericardial space. Thus, RV hypertrophy causes the septum to bow leftwards (Stool et al., 1974; Louie et al., 1986; Marcus et al., 2001; Gan et al., 2006; Hardziyenka et al., 2011), reducing LV diastolic loading and prompting the LV to reduce in mass and in cavity size, and hence to undergo atrophic remodeling (Hardziyenka et al., 2011; Meyer, 2014). One universal finding consistent with LV atrophic remodeling is that the LV is “under-filled.” This is demonstrated in RVF patients where the LV filling rate (Louie et al., 1986; Marcus et al., 2001) and stroke volume are reduced (Marcus et al., 2001; Gan et al., 2006; Hardziyenka et al., 2011, 2012). This is also observed in experimental data obtained from a widely-studied rat model of pulmonary hypertensive RVF induced by a single injection of monocrotaline, which damages the pulmonary vascular cell linings (Hardziyenka et al., 2011, 2012). Using the same rat model, LV systolic pressure was shown to be reduced (Akhavein et al., 2007; Correia-Pinto et al., 2009; Hadi et al., 2011; Fontoura et al., 2014).

Nevertheless, it remains unclear whether LV systolic impairment is a direct mechanical effect of the pressure-overloaded and hypertrophic RV impinging on the septum wall or a longer-term atrophic structural remodeling response. We hypothesize that LV systolic impairment is largely due to the acute effect of RV hypertrophy-induced reduction in diastolic filling whereas the chronic response of structural remodeling plays only a minor role. This hypothesis stems from three supporting lines of evidence. First, LV mechanical function returns to normal upon relief of RV pressure overload. In patients with chronic thromboembolic pulmonary hypertension following pulmonary endarterectomy (Dittrich et al., 1989; Menzel et al., 2000; Hardziyenka et al., 2011; Mauritz et al., 2012), and, in patients with pulmonary stenosis (Lurz et al., 2009) following percutaneous pulmonary valve implantation, LV systolic and diastolic functions have been shown to be improved and even normalized. Normalization was achieved in relatively short periods - within 2 weeks (Menzel et al., 2000) or even within 1 week (Dittrich et al., 1989) after relieving the RV from pressure overload. Second is evidence from LV muscle strips dissected from the monocrotaline-treated rat. Dissected LV muscle strips have been completely freed from the effect of RV pressure overload. Their developed stress was reported to be normal (Kögler et al., 2003), suggesting that the intrinsic mechanical function of the LV myocardium is not affected by PAH. Third, evidence arises from examination of histological data which showed normal collagen content (Honda et al., 1992; Ishikawa et al., 1992; Hardziyenka et al., 2012) and absence of tissue fibrosis (Lourenço et al., 2006; Fowler et al., 2015) in LV myocardial samples dissected from RVF hearts.

To test our hypothesis, we studied the LV of the monocrotaline-induced PAH rat heart in the presence or absence of the effect of RV pressure overload on the LV. Experiments were performed across multiple-scales: the whole heart *in vivo* and *ex vivo*, and its trabeculae *in vitro*.

## Materials and methods

Experiments were conducted in accordance with protocols approved by The University of Auckland Animal Ethics Committee.

### Animal preparation

Male Wistar rats (9-10 weeks old, 300-325 g) were divided into two groups. The RVF group received a single subcutaneous injection of monocrotaline (60 mg/kg), whereas the Control group received a comparable volume of saline vehicle. Rats were weighed three times a week up to Week 5 and daily thereafter as the RVF cohort was expected to show signs of right-heart failure: dyspnea, consecutive days of weight loss, and piloerection. Either upon showing these signs, or at Week 6, the RVF rats were used for energetics experiments, along with an age-matched animal from the Control group.

### Longitudinal measurements of *in vivo* hemodynamics

A separate group of Wistar rats underwent surgery for measurement of arterial blood pressure. Detailed surgical procedures have been published previously (Guild et al., 2015). Briefly, under isofluorane anesthesia, a telemetric catheter (TRM54P, Millar Inc, Houston, TX) was placed in the abdominal aorta, and secured with tissue adhesive and a mesh patch. The body of the telemeter was placed inside the abdomen and secured to the ventral wall. Rats were given prophylactic antibiotics and analgesia before and following surgery. They were individually housed in a standard rat cage placed on a TR180 SmartPad (Millar Inc, Houston, TX) which acted as a wireless battery charger and receiver. Arterial pressure signals were recorded in LabVIEW, sampled at 500 Hz and the averages of the derived heart rates and mean arterial, systolic, and diastolic blood pressures saved every 2 s. The implanted rats were given at least 1 week to recover from the surgery, following which a further week of baseline recording was performed before they were administered either monocrotoline (RVF group) or an equivalent volume of saline via subcutaneous injection (Control group). Monitoring continued for 6 weeks post-injection. In total, continuous recordings were achieved in 8 RVF and 6 Control rats.

### *In vivo* measurements of ventricular pressures at sacrifice

Approximately 6 weeks post-injection, all implanted rats were anesthetized using isoflurane (2% in oxygen), intubated and ventilated. Arterial pressure and heart rate were monitored throughout via the implanted telemeter. Incisions were made in the chest and diaphragm to allow insertion of a pressure-volume catheter (SPR-838, Millar Inc) into the left ventricle, and a second pressure-volume catheter (model SPR-869, Millar Inc) into the right ventricle. Simultaneous measurement was not possible due to interference between the two catheters. Hence, the order of ventricular recordings was randomized. Data were acquired for computer analysis using the LabChart 7 software system (Powerlab, ADInstruments).

### *Ex vivo* working-heart experiments

Each rat was deeply anesthetized with isoflurane, and its body mass measured prior to injection with heparin (1,000 IU/kg). Following cervical dislocation, the heart (and lungs) were excised and plunged into chilled Tyrode solution. The aorta was immediately cannulated for Langendorff perfusion at 70 mmHg with oxygenated Tyrode solution at room temperature. The solution contained, in mmol L^−1^, 130 NaCl, 6 KCl, 1 MgCl_2_, 1.5 CaCl_2_, 0.5 NaH_2_PO_4_, 10 Hepes and 10 glucose, and was pH adjusted to 7.4 using Tris.

While submerged under Tyrode solution, all vessels of the continuously-perfused heart were ligated, except for the pulmonary artery and one of the pulmonary veins, both of which were cannulated. Oxygen sensors, with their associated temperature probes, were inserted into the aortic cannula (to reside just distal to the coronary ostia) and the pulmonary artery to measure the partial pressure of oxygen in solution before and after flowing through the coronary vasculature. Flow probes, connected to flow meters, measured the rates of aortic and coronary outflow. The fully-cannulated heart was then attached to the working-heart rig (Goo et al., 2013, 2014b; Loiselle et al., 2014) and enclosed by a water-jacketed glass chamber equilibrated to 37°C. Intrinsic heart rate was measured, followed by external pacing at 5 Hz, by placing a stimulus electrode at the sinoatrial node. A pressure-volume catheter was inserted into the left ventricle via the pulmonary vein cannula.

Perfusion was then switched to working-heart mode. The left ventricle was subjected to a fixed preload of 17 mmHg while 8-10 different afterloads were presented in random order, ranging from 30 mmHg (at which point the coronary flow was near zero) to maximal (typically 100 mmHg, at which point the aortic flow was near zero). LV afterload was altered by changing the height of the aortic tubing (Goo et al., 2013, 2014a,b; Loiselle et al., 2014). To remove the effect of RV pressure overload, RV afterload was fixed at a low value (10 mmHg) and no Tyrode solution was allowed to flow into the RV except the coronary effluent from the LV via the coronary sinus. Mechanical work output of the heart, consisting of the sum of contributions from both ventricles, was normalized to heart wet mass. LV work was calculated by multiplying LV afterload (aortic pressure) and LV stroke volume (estimated from the sum of aortic and coronary effluent), whereas RV work was calculated by multiplying RV afterload and RV stroke volume (consisting of the coronary effluent only). RV work contributed, on average, 5% of the total work output of the heart, for both rat groups. Oxygen consumption of the heart, expressed per wet mass, was calculated from the product of coronary flow, the arterio-venous difference in partial pressure of oxygen and the solubility of oxygen in saline. Oxygen consumption was corrected for the trans-epicardial loss of oxygen (on average, 1%) across the epicardial surface to the surrounding air (Goo et al., 2013; Han et al., 2014a). Efficiency was calculated as the ratio of mechanical work output to the energetic equivalent of oxygen consumption.

### *In vitro* trabecula experiments

Immediately following the whole-heart experiments, the heart was detached from its cannulae, and perfused with Tyrode solution containing 20 mmol L^−1^ butanedione monoxime (BDM) and low Ca^2+^ (0.3 mmol L^−1^). Submerged under the same solution, trabeculae were excised from the left ventricle. A geometrically-suitable trabecula was transferred to, and mounted in, a work-loop calorimeter (Taberner et al., 2011). A total of 14 Control trabeculae and 20 RVF trabeculae were studied. There were no differences in the dimensions of either length (3.44 ± 0.17 mm and 3.51 ± 0.13 mm) or cross-sectional area (0.062 ± 0.011 mm^2^ and 0.078 ± 0.009 mm^2^), as calculated from two orthogonal estimates of diameters (minor axis: 253 ± 25 μm and 274 ± 14 μm; major axis: 283 ± 23 μm and 345 ± 23 μm).

In the calorimeter, with improved thermal resolution for experiments at body temperature (Johnston et al., 2015; Taberner et al., 2015), the trabecula was superfused with the same Tyrode solution as had been used for the working-heart-−1.5 mmol L^−1^ [Ca^2+^]_o_ but without BDM. The ends of the trabecula were held by platinum hooks for length control (upstream) and force measurement (downstream). The heat output of the trabecula was estimated from the flow rate-dependent temperature sensitivity and the increase in temperature of the superfusate as measured by the upstream and downstream arrays of thermopiles. Superfusate flow rate was electronically maintained at 0.55 μL s^−1^ for optimal thermal sensitivity (Johnston et al., 2015) and negligible risk of inducing an hypoxic core (Han et al., 2011). The trabecula was stimulated via platinum electrodes to contract at 4 Hz. It was then gradually stretched to optimal length (*L*_*o*_) to achieve maximal active force development. The entire calorimeter system was then optically-isolated and thermally-insulated in its enclosure. By controlled heating of the vibration-isolated optical table on which the entire calorimeter system was mounted, the temperature within the enclosure was electronically maintained at 37°C.

Firstly, the trabecula contracted isometrically while its force, length, and rate of suprabasal heat production were simultaneously measured. Upon reaching steady states of isometric force and heat-rate, it was then subjected to work-loop contractions at a user-specified afterload and reaching steady state before switching back to isometric contraction. This procedure was repeated for six afterloads, ranging from maximal (isometric force) down to passive force. The stimulus was then halted, allowing force to return to its passive level and heat to return to suprabasal baseline. Secondly, stimulation was recommenced and the trabecula was again subjected to isometric contractions, but with its length progressively reduced in six steps from *L*_*o*_ to minimal length where developed force was near zero. Stimulus was halted between these length steps. Muscle stress was quantified by dividing force by cross-sectional area, whereas muscle length was expressed relative to *L*_*o*_. Muscle heat output was corrected for the thermal artifacts resulting from electrical stimulation (on average, 5%), quantified at the end of an experiment with the trabecula removed from the calorimeter. Muscle work was calculated by integrating stress as a function of relative muscle length over the entire period of the twitch. Change of enthalpy was given by the sum of work and suprabasal heat, and suprabasal efficiency was taken as the ratio of work to change of enthalpy (Han et al., 2012).

### Statistical analyses

Data from each heart or trabecula were fitted using polynomial regression up to the 3rd order. The resulting regression lines were averaged within groups using the Random Coefficient Model within Proc Mixed in the SAS statistical package, and tested for the statistical significance of differences between groups (*P* < 0.05). Graphical and tabulated values were expressed as mean ± standard error (SE), and for the latter, ANOVA was used to test for differences. In each plot, the means ± SEs were superimposed to demonstrate the greatest data variability.

## Results

### *In vivo* heart rate and blood pressures

**Figure 1:**
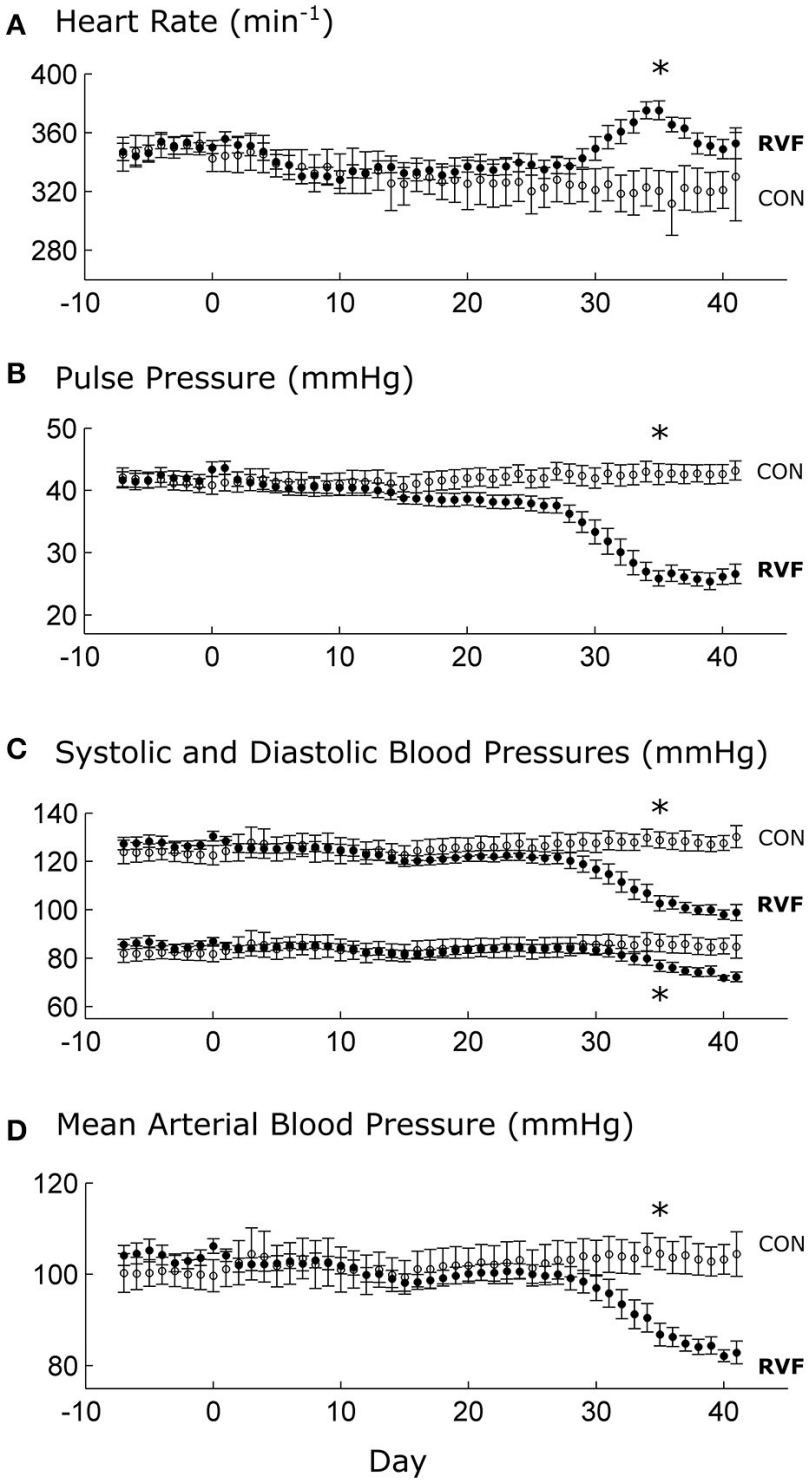
Mean ± SE *in vivo* heart rate and blood pressures of control and right-ventricular failing rats as functions of time. Day 0 indicates injection of either monocrotaline or saline vehicle, following a week of baseline recording. At Day 28, significant differences (^*^P < 0.05) between RVF rats (*n* = 8) and Control rats (*n* = 6) begin to be evident in heart rate **(A)**, pulse pressure **(B)**, and blood pressures **(C,D)**.

The heart rates and blood pressures of control and RVF animals were similar and remained relatively constant until approximately day 30 post-injection. After this time point, the *in vivo* heart rate of the RVF rats began to rise over a period of a week, before falling back again. This was accompanied by a sustained reduction of pulse pressure, systolic and diastolic blood pressures, and mean arterial pressure (Figure 1).

### *In vivo* heart rate and ventricular pressure at sacrifice

**Figure 2:**
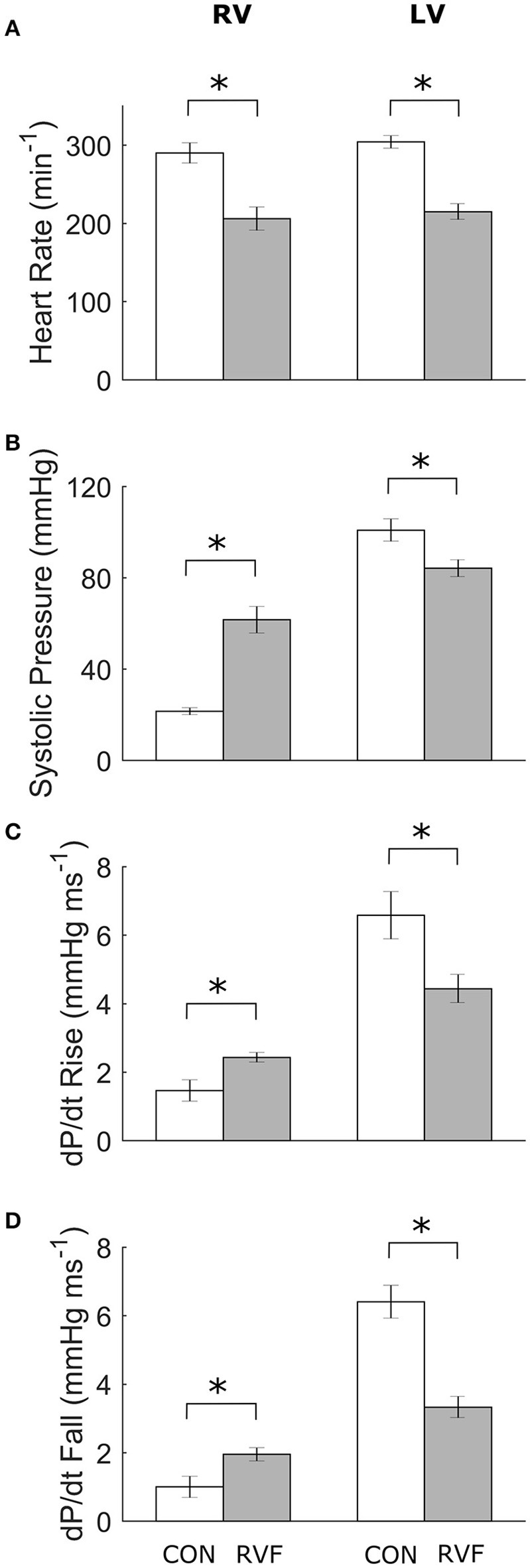
Mean ± SE *in vivo* heart rates and pressure of the ventricles of control and right-ventricular failing rats at Week 6. Data obtained from the right ventricle (4 Control and 5 RVF rats) and the left ventricle (5 Control and 7 RVF rats). Significant differences (^*^P < 0.05) between rat groups were observed in heart rate **(A)**, systolic ventricular pressure **(B)**, and the rates of rise **(C)** and fall **(D)** of ventricular pressure.

At the end of Week-6 post-injection, the *in vivo* heart rate was lower in the RVF rats (Figure 2A). They developed pulmonary hypertension, as evident by their greater right-ventricular systolic pressure (Figure 2B), associated with increased rates of rise (Figure 2C) and fall (Figure 2D) of pressure. The RVF rats showed lower left-ventricular systolic pressure (with lower rates of rise and fall), consistent with atrophic remodeling.

### Development of pulmonary hypertension

At sacrifice, the RVF rats showed clear signs of PAH with increased lung wet mass and heart wet mass (Table 1). The right ventricle had hypertrophied, as evident by an increase in both wall thickness and mass. In contrast, the left ventricle had atrophied, as evidenced by reduced wall thickness and mass.

**Table 1:** Morphometric characteristics of Control and RVF rats at sacrifice.

**Parameter**	**CON**	**RVF**
Body mass (g)	465 ± 6	394 ± 7^*^
Tibial length (mm)	43.7 ± 0.4	43.0 ± 0.3
**LUNG**		
Mass (g)	1.58 ± 0.03	2.49 ± 0.12^*^
Mass/body mass (%)	0.34 ± 0.01	0.64 ± 0.04^*^
Mass/tibial length (g m^−1^)	36.1 ± 0.7	57.7 ± 2.7^*^
**HEART**		
Mass (g)	1.39 ± 0.02	1.60 ± 0.03^*^
Mass/body mass (%)	0.30 ± 0.00	0.41 ± 0.01^*^
Mass/tibial length (g m^−1^)	31.8 ± 0.6	37.1 ± 0.8^*^
**RIGHT VENTRICLE**		
Wall thickness (mm)	1.52 ± 0.02	2.12 ± 0.06^*^
Wall thickness/heart mass (mm g^−1^)	1.10 ± 0.03	1.34 ± 0.05^*^
Mass (g)	0.26 ± 0.01	0.56 ± 0.01^*^
Mass/heart mass	0.19 ± 0.01	0.35 ± 0.01^*^
**LEFT VENTRICLE**		
Wall thickness (mm)	3.60 ± 0.06	3.41 ± 0.07^*^
Wall thickness/heart mass (mm g^−1^)	2.60 ± 0.06	2.17 ± 0.07^*^
Mass (g)	0.92 ± 0.03	0.81 ± 0.02^*^
Mass/heart mass	0.69 ± 0.01	0.51 ± 0.01^*^

*Values are means ± SE*.

* *P < 0.05. n = 24 for Control and n = 28 for RVF, except for RV mass and LV mass where n = 6 for Control and n = 11 for RVF*.

### Energetics of the heart *ex vivo*

**Figure 3:**
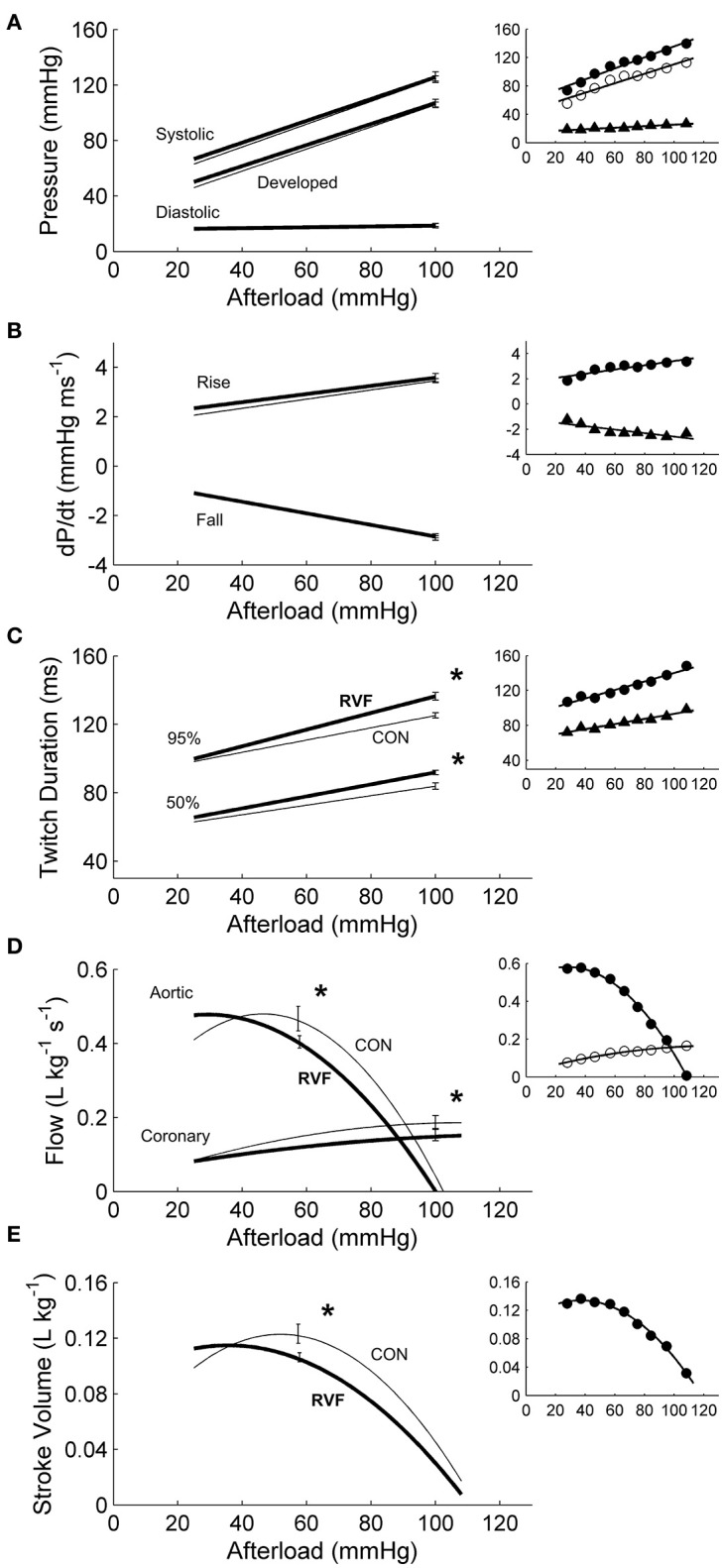
Mechanics and hemodynamics of *ex vivo* left ventricles of hearts of control and right-ventricular failing rats. There were no statistically significant differences of the average regression lines between the control hearts (thin lines) and the RVF hearts (thick lines) in the pressures **(A)** or in the maximal rates (dP/dt) of rise and fall of twitch stress **(B)**. However, as indicated by the asterisks, RVF hearts had greater average twitch durations **(C)**, and reduced aortic and coronary flows **(D)**, as well as reduced stroke volumes **(E)**. Developed pressure is the difference between systolic and diastolic pressure. Twitch duration is quantified at 95% and 50% of peak systolic pressure. Stroke volume is the sum of aortic and coronary flow rates. All insets display representative data set obtained from a single representative heart.

**Figure 4:**
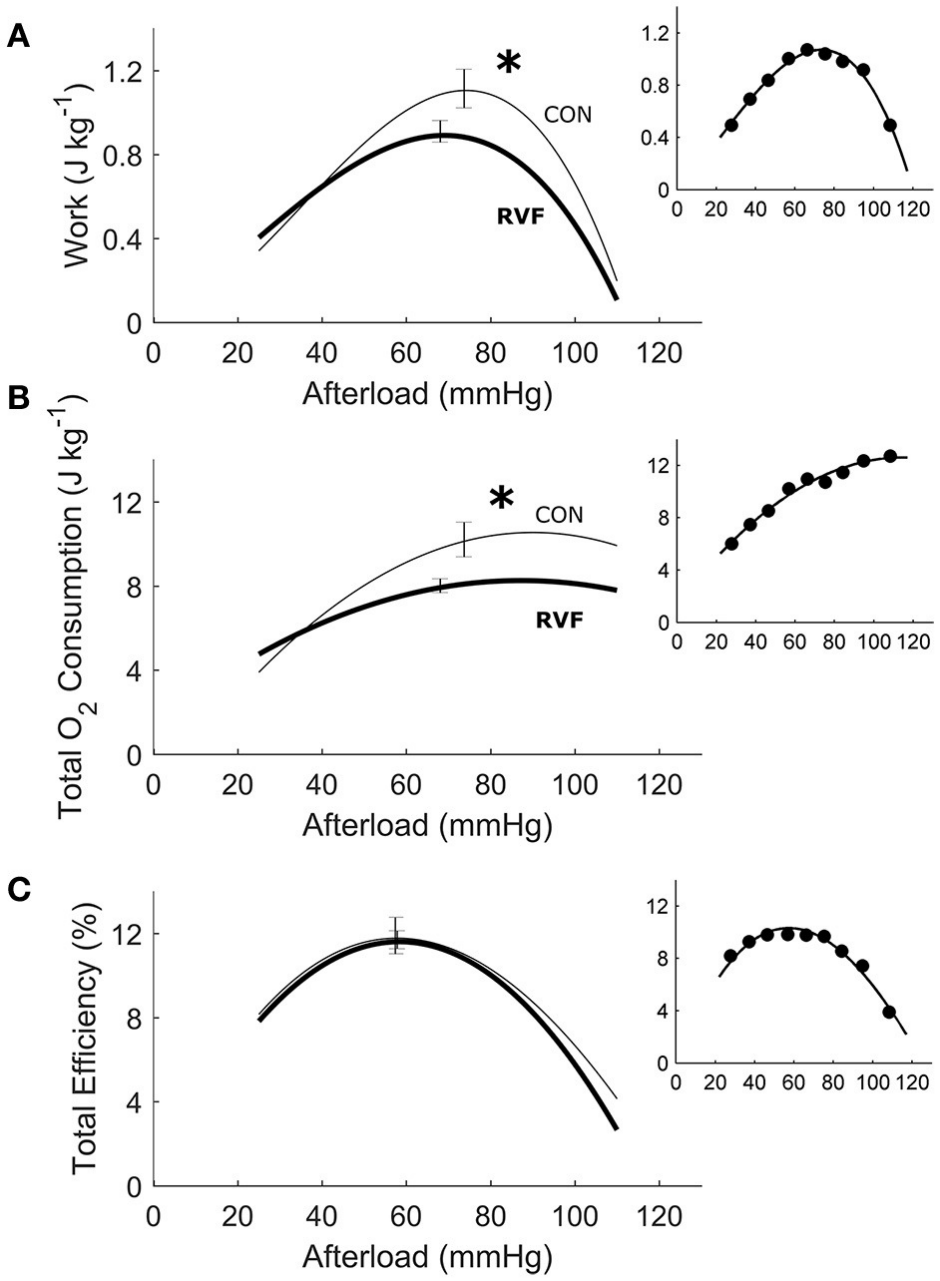
Energetics of *ex vivo* left ventricles of hearts from control and right-ventricular failing rats. The two rat groups have the same total efficiency over a wide range of afterloads **(C)**, given that the average reduction in mechanical work **(A)** in the RVF hearts was offset by a comparable reduction in total oxygen consumption **(B)**. Asterisks indicate statistical significant differences between groups. All insets display data obtained from the same representative heart as shown in Figure 3.

In total, 13 Control hearts and 21 RVF hearts were studied in the working-heart rig. The RVF hearts had a lower *ex vivo* heart rate (2.8 ± 0.1 Hz vs. 3.8 ± 0.1 Hz). To accommodate this difference in *ex vivo* heart rate, both groups of hearts were externally paced at 5 Hz. The amplitudes and the rates of pressure developed within the left ventricle were not different between groups (Figures 3A,B), but the duration of pressure developed was greater in the RVF rats, particularly at high afterloads (Figure 3C). Both aortic and coronary outflows, as well as their sum, which denotes stroke volume, were lower in the RVF rats (Figures 3D,E). The lower stroke volume in the RVF rats gave rise to reduced mechanical work output (Figure 4A), along with a proportional reduction of oxygen consumption (Figure 4B), hence total efficiency was not different to that of Control rats (Figure 4C).

### Energetics of the trabeculae *in vitro*

**Figure 5:**
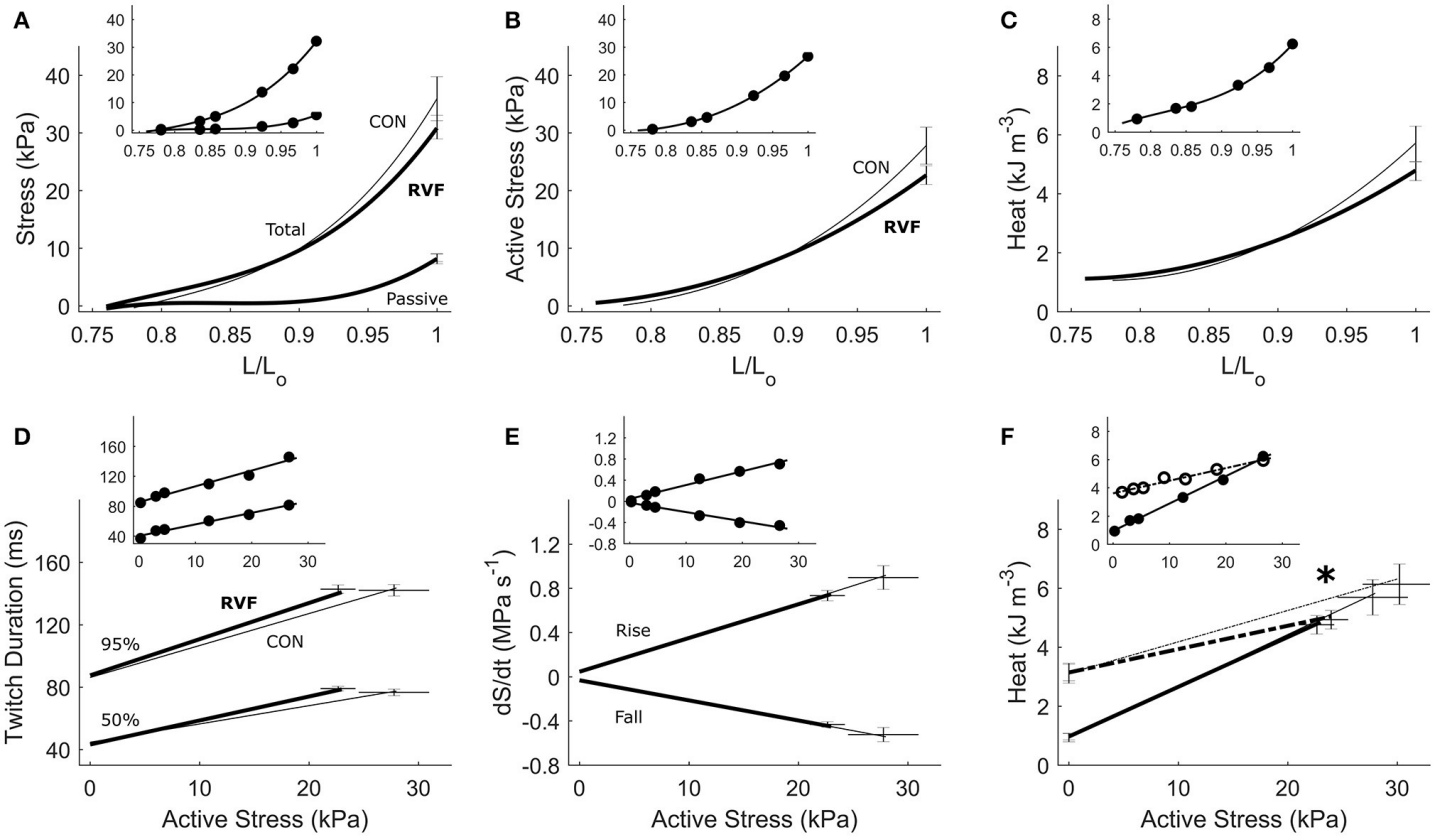
Energetics of *in vitro* left-ventricular trabeculae contracting isometrically. Active stress **(B)** is the difference between total stress and passive stress **(A)**. It, and heat output **(C)**, as functions of relative length, were not different between the control (thin lines) and RVF trabeculae (thick lines). Likewise, no difference between groups was seen in twitch duration **(D)** or rates of rise and fall of the twitch (*dS/dt*) **(E)**. Suprabasal heat as a function of active stress was also not different between groups **(F)**. The dotted lines in **(F)** (and dotted circles in its inset) represent the heat-stress relations from the work-loop (shortening) protocol, showing a lower slope in the RVF group vs. the Control group (as indicated by the asterisk). All insets display data obtained from a representative trabecula.

Under the isometric protocol, there was no difference in energetics between RVF trabeculae and Control trabeculae, as functions of muscle length. Likewise there were no differences in twitch characteristics, in terms of amplitude (Figures 5A,B), duration (Figure 5D) or rates of rise and fall of stress (Figure 5E). The suprabasal heat output, either as a function of length (Figure 5C) or of active stress (Figure 5F), also did not differ between groups. Neither were the heat-intercepts of the heat-stress relations under either isometric or work-loop protocols different between groups. In Figure 5F, the slopes of the heat-stress relations under the work-loop protocol (broken lines) were lower in the RVF group.

**Figure 6:**
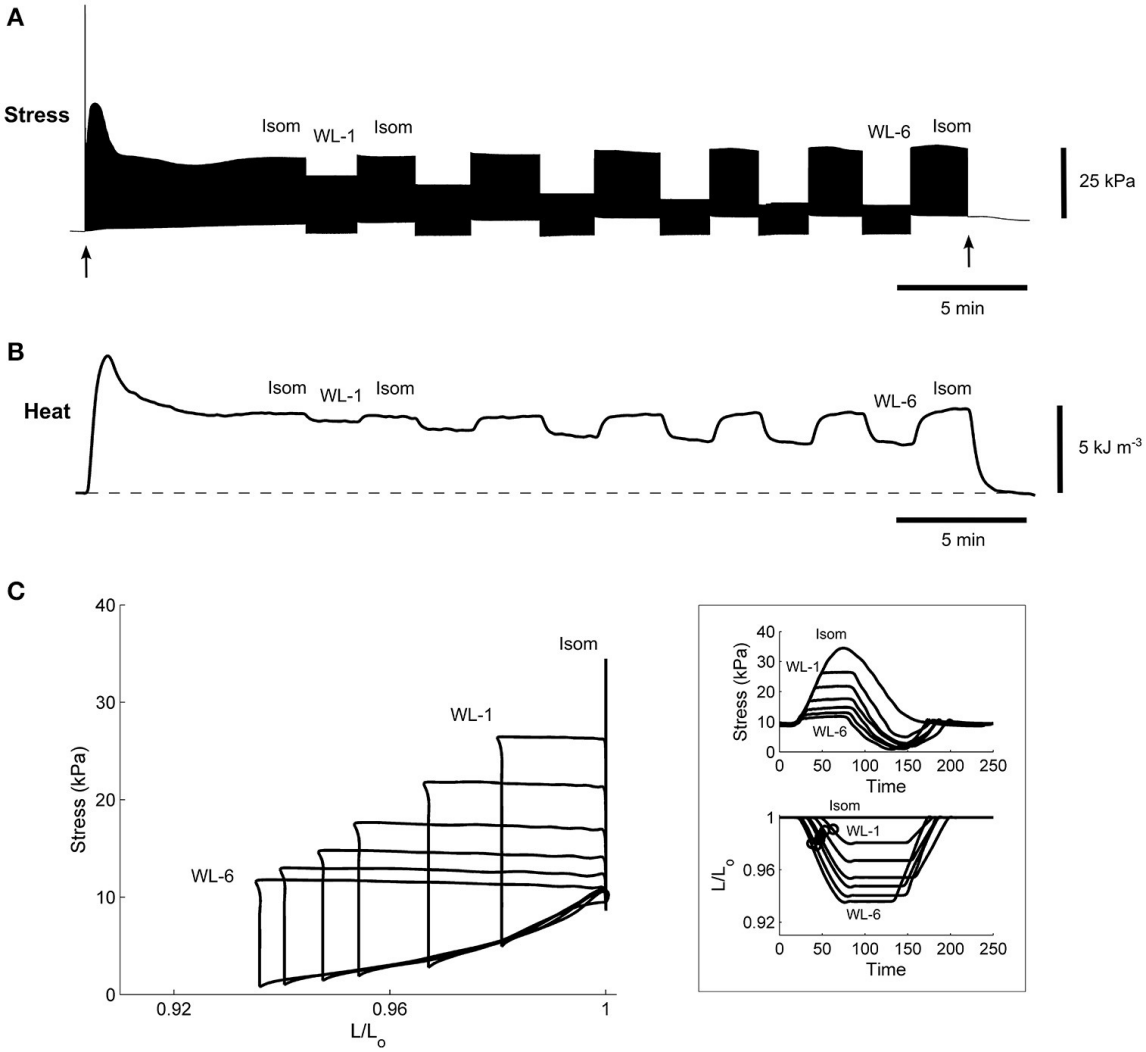
A representative experimental record of a single trabecula undergoing work-loop contractions. Stimulation started at the time indicated by the first arrow and halted at the second arrow. The trabecula underwent a series of work-loop contractions at 6 different afterloads (labeled “WL-1” and “WL-6”) until steady-state (~2 min) before and after an isometric contraction (labeled “Isom”), where its stress production **(A)**, suprabasal heat output **(B)**, and length change (not shown) were simultaneously recorded. Steady-state work-loops resulted from plotting stress as a function of length **(C)**. The inset in **(C)** shows the steady-state twitch-time profiles (upper panel) and corresponding length changes (lower panel), where the open circles indicate the maximal slopes during the shortening phase and are an index of velocity of shortening.

**Figure 7:**
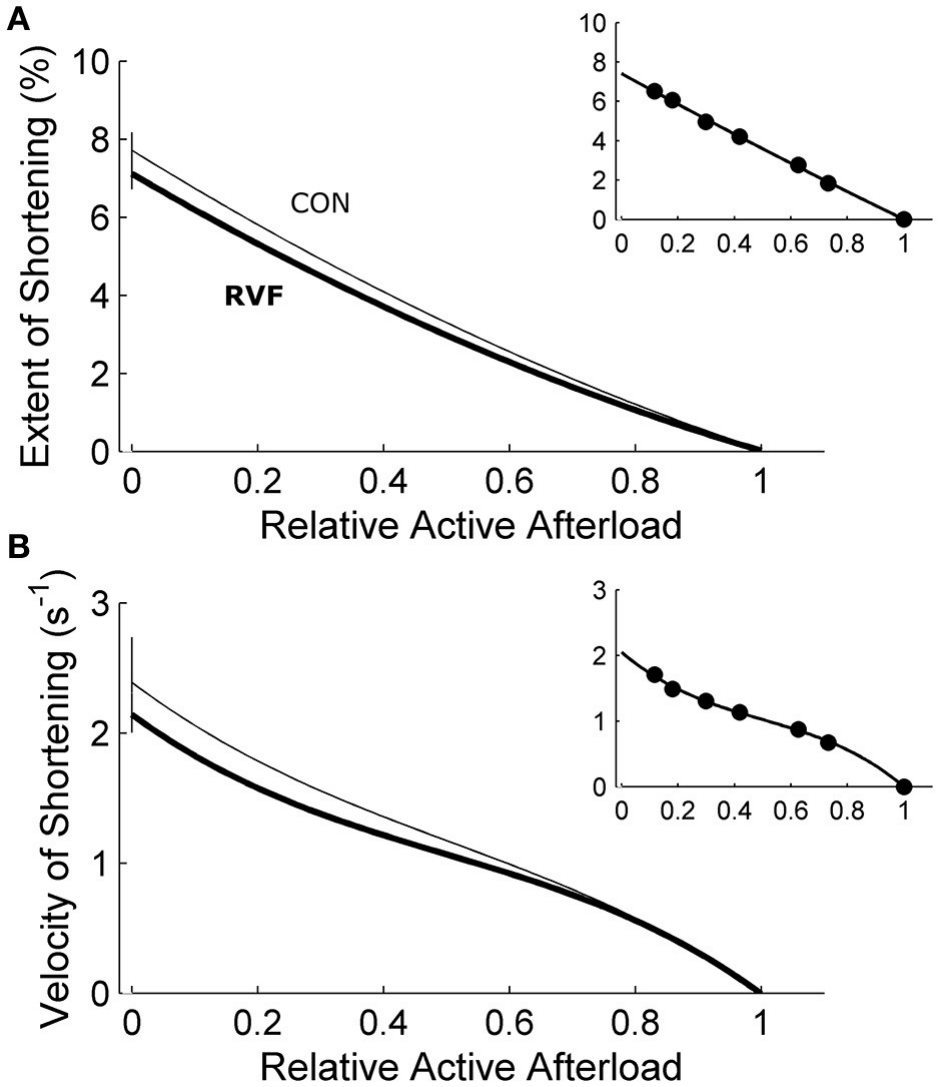
Shortening of in vitro left-ventricular trabeculae during work-loop contractions. Extent of shortening **(A)** (calculated as the widths of the loops in Figure 6C) and maximal velocity during shortening **(B)** (calculated as the maximal slope of length change during the isotonic shortening phase of the loop; see the inset of Figure 6C) were not different between the control trabeculae (thin lines) and the RVF trabeculae (thick lines). The insets display data obtained from a representative trabecula.

**Figure 8:**
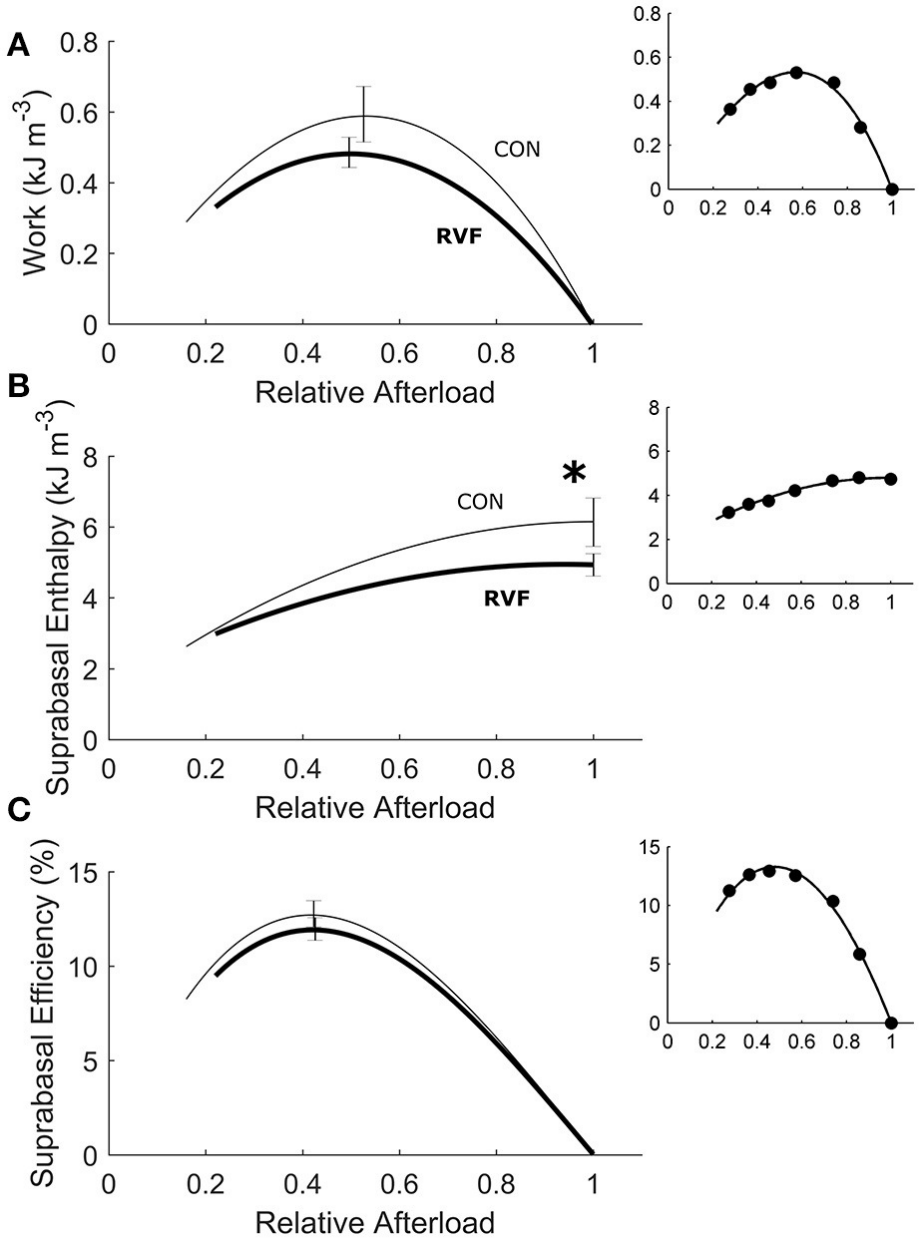
Energetics of *in vitro* left-ventricular trabeculae performing work-loop contractions. As functions of relative afterload, work output **(A)** (equivalent to the area of the loop in Figure 6C) was not statistically different between groups (*P* = 0.2090) but change of enthalpy **(B)** (the sum of work and suprabasal heat output) was lower in the RVF trabeculae (thick lines) than in the Control trabeculae (thin lines), as indicated by the asterisk. Suprabasal efficiency was not different between groups **(C)**. All insets show data set obtained from a representative trabecula.

Under variably-afterloaded work-loop contractions (Figure 6), the RVF trabeculae showed no differences in the extent of shortening (the width of the loop) or in the velocity of shortening with respect to the Control trabeculae (Figure 7). Given that the stress production and the widths of the work-loop were not different between groups, mechanical work output (the area of the loop) was not significantly different (Figure 8A). However, change of enthalpy was significantly lower in the RVF trabeculae (Figure 8B). Suprabasal efficiency did not differ between groups (Figure 8C).

## Discussion

To our knowledge, this study represents the first to have investigated the energetic performance of the LV in PAH-induced RV failure across three physiological scales: the *in vivo* heart, the *ex vivo* working-heart and *in vitro* trabeculae. Our *in vivo* experiments clearly demonstrated LV atrophic remodeling, as evidenced by reduced ventricular mass and wall thickness (Table 1), as well as a sustained reduction of systemic blood pressure (Figure 1) in RVF rats. The *in vivo* experiments also revealed LV systolic dysfunction, as evidenced by a lower ventricular pressure (Figure 2). Our *ex vivo* and *in vitro* experiments allowed removal of the effect of pressure overload of the RV on LV. These experiments revealed that the LV was capable of developing systolic pressure as high as that of the healthy heart (Figure 3). Likewise, the LV trabeculae *in vitro* were able to develop a normal stress (Figure 5). In both cases, LV energy efficiency was unaffected (Figures 4, 8). These results show that reduced LV systolic pressure can be restored immediately by removing the effect of RV pressure overload and that the effect of LV atrophic remodeling is limited to reducing the LV stroke volume.

### Heart rates *in vivo* and *ex vivo*

Commencing at Week 4 post-injection of monocrotaline, the RVF rat showed reduced LV workload, as evidenced by the reductions of both pulse and mean arterial pressures (Figure 1). These reductions in systemic blood pressures were associated with a temporary elevation of heart rate, which was observed in all RVF rats. Increased heart rate in PAH has been observed in human patients (Marcus et al., 2001; Gan et al., 2006; Hardziyenka et al., 2011; Manders et al., 2014) where it is seen to vary with the severity of hypertension (Wong et al., 2011), as well as in monocrotaline rats 4-week post-injection (Sanyal et al., 2012).

We interpret the increase in heart rate as a reflex response of the sympathetic nervous system (Sanyal et al., 2012). An increase in heart rate is necessary to compensate for the drop in LV stroke volume (Marcus et al., 2001; Gan et al., 2006; Hardziyenka et al., 2011; Manders et al., 2014) in order to maintain cardiac output (Stool et al., 1974). Our results show that this reflex response was only transient as heart rate rose in Week 5 but fell during Week 6, despite a sustained drop in systemic blood pressure. These results may explain, in terms of progression of the disease, why some studies show no effect of PAH on heart rate in the human patient (Krayenbuehl et al., 1978) and in the monocrotaline rat (Wong et al., 2011).

At Week 6 post-injection, we measured a 30% lower *in vivo* heart rate in the deeply anesthetized RVF rats relative to Control (Figure 2A), consistent with the results of Correia-Pinto et al. (2009) at the same time point of progression of PAH. Our results from anesthetized rats are at odds with those measured in conscious rats at the same time point (Figure 1). Not only was the heart rate lower in the anesthetized state in both groups, the heart rate was also lower in the anesthetized RVF rats. These findings may reflect a synchronous effect of attenuation of sympathetic drive and elevation of parasympathetic nerve activity in the anesthetized state, and this synchronous effect appears to be more pronounced in the RVF rat. We also saw a comparable 30% lower *ex vivo* heart rate in the RVF hearts compared with the Control hearts when mounted in the working-heart rig.

### Pressure and stress development

The RVF rats had a lower *in vivo* heart rate (200 vs. 300 beats/min). Their RV developed greater systolic pressure along with greater *dP/dt* rise and fall (Figure 2). These findings are in accord with many (Falcão-Pires et al., 2009; Wang et al., 2011) but not all (Hessel et al., 2006) studies of the monocrotaline-treated rat. Conversely, in the LV, systolic pressure was reduced and was accompanied by reduced rates of ±*dP/dt*, results that are consistent with previous studies using the same animal model (Akhavein et al., 2007; Correia-Pinto et al., 2009; Hadi et al., 2011; Fontoura et al., 2014).

In our *ex vivo* working-heart experiments, we externally paced hearts to beat at 5 Hz for both rat groups. The RV workload was set to be negligible compared with that of the LV. Under these conditions, the LV of the RVF heart was able to generate the same systolic pressure and ±*dP/dt* as the control heart across a range of afterloads (Figure 3). Likewise, both groups of trabeculae *in vitro*, contracting at 4 Hz, were capable of developing the same stress (S) and ±*dS/dt* across a range of active stress (Figure 5). These findings suggest that, upon being freed from the effects of RV pressure overload, the LV of the RVF rat is able of developing normal ventricular pressure and tissue stress. These results are consistent with those of Kögler et al. (2003), studying the same rat model, where their ventricular muscle strip preparations were stimulated at a range of frequencies (1–7 Hz).

For a given pressure or stress, twitch duration was greater in the atrophied LV of the RVF heart (Figure 3C), but its prolongation was much less in LV trabeculae (Figure 5D). Prolongation of twitch duration of the atrophied LV is a feature consistently reported in the literature using the monocrotaline rat model (Lourenço et al., 2006; Correia-Pinto et al., 2009; Falcão-Pires et al., 2009), as well as in the hypertrophied LV myocardium induced by diabetes (Han et al., 2014c) and systemic hypertension (Han et al., 2014b). This effect is linked to electrical abnormalities, specifically, prolongation of the action potential, in both monocrotaline-treated rats (Benoist et al., 2011; Hardziyenka et al., 2012) and human patients (Louie et al., 1986; Hardziyenka et al., 2012). Prolongation of action potential has been attributed to a reduction of the voltage-gated transient outward K^+^ current (Benoist et al., 2011; Hardziyenka et al., 2012).

### Stroke volume and shortening

Reduced LV stroke volume has been consistently reported in the literature using the same animal model (Hardziyenka et al., 2012) as well as in PAH patients (Marcus et al., 2001; Gan et al., 2006). Those results were both obtained in the presence of RV pressure overload. In our *ex vivo* working-heart experiments, the RV afterload was set relatively low to diminish its effect on LV hemodynamic performance. We observed normalization of LV pressure but not LV stroke volume. *Ex vivo* RVF hearts discharged reduced aortic and coronary flows (Figure 3). These reductions mean that LV stroke volume (the sum of both aortic and coronary outflows), and hence cardiac output, are reduced. Our explanation for this finding resides largely in structural remodeling in both ventricles and, to a lesser extent, in the prolongation of LV twitch duration in the RVF heart (Figure 3C). Both groups of hearts were stimulated to beat at the same rate, and hence a prolongation of twitch duration dictates an abbreviation of the diastolic filling duration. Given its inherently reduced rate of LV diastolic filling (Marcus et al., 2001; Hardziyenka et al., 2011), coupled with reduced diastolic period, the RVF hearts thus have a lower LV stroke volume.

The shift of myosin heavy chain (MHC) from the fast to the slow isoforms could also explain our findings of prolongation of twitch duration. Several histological studies show a positive effect of monocrotaline on the percentage of the beta isoform of the MHC protein (Ishikawa et al., 1992; Correia-Pinto et al., 2009) in rat homogenized LV tissues, or a negative effect on the expression of alpha-MHC isoform in the LV of *in situ* hybridized rat heart sections (Hardziyenka et al., 2011). Despite these documented isoenzymatic shifts of MHC from the fast toward the slow isoforms, we found no significant change in the stress-velocity relationship of the LV trabeculae dissected from the RVF heart (Figure 7B). We are unaware of any study that has quantified velocity of shortening in the rat LV tissue of monocrotaline-induced PAH, but note that the peak velocity of contractile element shortening, as well as the mean velocity of LV circumferential fiber shortening, remain unchanged in chronic PAH human patients (Krayenbuehl et al., 1978).

In our experiments, we quantified the active deformation of LV trabeculae dissected from RVF hearts as the maximum extent of shortening in work-loop contractions. Our data showed no change in the extent of shortening between the two groups (Figure 7A), suggesting that the intrinsic contractile properties of LV tissues from RVF hearts, revealed in the absence of RV pressure overload, are unaffected. These results are consistent with the experiments performed by Stool et al. (1974) where acutely decreasing PAH in canine hearts increased LV strain. Our results are also supported by studies showing negligible effects of PAH on either collagen content (Honda et al., 1992; Ishikawa et al., 1992; Hardziyenka et al., 2012) or extent of fibrosis (Lourenço et al., 2006; Fowler et al., 2015) of LV tissues, although several studies have shown a positive effect (Lamberts et al., 2007; Hadi et al., 2011).

### Maintenance of energy efficiency

Despite removal of the effect of RV pressure overload, the work density of the LV remained lower in RVF hearts (Figure 4). Their lower work density arises from reduced aortic and coronary flows, and hence stroke volume. We attribute this to the atrophic response, which reduced the mass of the LV. The reduction in work density is accompanied by a concomitantly lower total oxygen consumption such that total energy efficiency of the RVF heart remained unchanged at a peak value around 12%. Our peak total efficiency value is consistent with those reported by Neely et al. (1967) who developed the first working rat heart preparation, the technique of which has largely been replicated in this study. With a relatively small RV work output, the isolated working rat heart preparation is specifically designed for assessing the LV energetic performance. Given the relatively large LV mass to heart mass ratio for our Control hearts (0.69 vs. 0.19 RV; Table 1), it is reasonable to normalize LV work output and heart oxygen consumption to the entire heart mass, as done by Neely et al. (1967), as well as by Han et al. in the study of LV hypertrophy (Han et al., 2014a, 2015). In contrast, the LV mass to heart mass ratio of the RVF hearts was reduced to 0.51, and that of RV was increased to 0.35 (Table 1). Given the larger RV mass of the RVF hearts, it is nevertheless still appropriate to normalize total work and oxygen consumption to the entire whole-heart mass for three reasons. First, we obtained consistent results between isolated LV trabeculae (Figure 8), and whole-hearts (Figure 4). Second, we obtained no differences between the two groups in work and oxygen consumption without normalization. Third, we also obtained no differences between the two groups in work and oxygen consumption with normalization to LV mass alone (results not shown).

The heat-stress relation (Figure 5F) arising from the isometric protocol provides an estimate of the stress-independent activation heat (Pham et al., 2017), and its difference to that of the work-loop protocol yields a measure of crossbridge shortening heat (Tran et al., 2017). The lower oxygen consumption and enthalpy output in the LV of the RVF group is not due to the heat of activation, as indicated by an absence of difference between groups in the heat-intercept of the isometric heat-stress relation. Instead, the lower oxygen consumption and enthalpy output arises from a lower heat output from the hydrolysis of ATP by cycling crossbridges particularly during shortening and at high active stresses, as the work-loop heat-stress relation is lower in the LV trabeculae of the RVF group.

## Conclusions

In PAH, the atrophied LV is capable of immediately regaining normal pressure and stress development upon relief of the effects of the pressure overloaded RV. However, the effects of reduced geometry of the atrophied LV remained: reduced LV stroke volume and, hence, stroke work density, as well as reduced oxygen consumption or heat output. Collectively, these findings suggest that reduced LV systolic pressure *in vivo* is largely due to the mechanical interference of RV pressure overload, whereas the reduced LV stroke work is a consequence of LV atrophic remodeling. Nevertheless, the atrophied LV maintains its energy efficiency, at both whole-heart and tissue levels. Our results explain the outcome of a rapid improvement of LV systolic function observed in patients with chronic pulmonary hypertension following surgical relief of RV pressure overload.

## Author contributions

Designed and performed the *ex vivo* experiments: J-CH, the *in vitro* experiments: J-CH, TP, and the *in vivo* experiments: S-JG. Animal preparation: LN. Data acquisition, J-CH, S-JG, and AT. Data analysis: J-CH, S-JG, TP, and DL. Data interpretation: J-CH, S-JG, TP, LN, KT, AT, and DL. All authors reviewed and approved the final version of the manuscript.

### Conflict of interest statement

The authors declare that the research was conducted in the absence of any commercial or financial relationships that could be construed as a potential conflict of interest.

## References

[B1] Akhavein F. St-Michel E. J. Seifert E. Rohlicek C. V. (2007). Decreased left ventricular function, myocarditis, and coronary arteriolar medial thickening following monocrotaline administration in adult rats. J. Appl. Physiol. 103, 287–295. 10.1152/japplphysiol.01509.2005 17412785

[B2] Benoist D. Stones R. Drinkhill M. Bernus O. White E. (2011). Arrhythmogenic substrate in hearts of rats with monocrotaline-induced pulmonary hypertension and right ventricular hypertrophy. Am. J. Physiol. Heart Circ. Physiol. 300, H2230–H2237. 10.1152/ajpheart.01226.2010 21398591 PMC3119089

[B3] Correia-Pinto J. Henriques-Coelho T. Roncon-Albuquerque R. Jr. Lourenço A. P. Melo-Rocha G. Vasques-Nóvoa F.. (2009). Time course and mechanisms of left ventricular systolic and diastolic dysfunction in monocrotaline-induced pulmonary hypertension. Basic Res. Cardiol. 104, 535–545. 10.1007/s00395-009-0017-3 19288153

[B4] Dittrich H. C. Chow L. C. Nicod P. H. (1989). Early improvement in left ventricular diastolic function after relief of chronic right ventricular pressure overload. Circulation 80, 823–830. 10.1161/01.CIR.80.4.823 2791245

[B5] Falcão-Pires I. Gonçalves N. Henriques-Coelho T. Moreira-Gonçalves D. Roncon-Albuquerque R. Leite-Moreira A. F. (2009). Apelin decreases myocardial injury and improves right ventricular function in monocrotaline-induced pulmonary hypertension. Am. J. Physiol. Heart Circ. Physiol. 296, H2007–H2014. 10.1152/ajpheart.00089.2009 19346461

[B6] Fontoura D. Oliveira-Pinto J. Tavares-Silva M. Leite S. Vasques-Nóvoa F. Mendes-Ferreira P.. (2014). Myocardial and anti-inflammatory effects of chronic bosentan therapy in monocrotaline-induced pulmonary hypertension. Rev. Port. Cardiol. 33, 213–222. 10.1016/j.repc.2013.09.016. 24780128

[B7] Fowler E. D. Benoist D. Drinkhill M. J. Stones R. Helmes M. Wüst R. C. I.. (2015). Decreased creatine kinase is linked to diastolic dysfunction in rats with right heart failure induced by pulmonary artery hypertension. J. Mol. Cell. Cardiol. 86, 1–8. 10.1016/j.yjmcc.2015.06.016 26116865 PMC4564291

[B8] Gan C. T.-J. Lankhaar J.-W. Marcus J. T. Westerhof N. Marques K. M. Bronzwaer J. G. F.. (2006). Impaired left ventricular filling due to right-to-left ventricular interaction in patients with pulmonary arterial hypertension. J. Physiol. Heart Circ. Physiol. 290, H1528–H1533. 10.1152/ajpheart.01031.2005 16284226

[B9] Goo S. Han J.-C. Nisbet L. A. LeGrice I. J. Taberner A. J. Loiselle D. S. (2014a). Dietary pre-exposure of rats to fish oil does not enhance myocardial efficiency of isolated working hearts or their left ventricular trabeculae. J. Physiol. 592, 1795–1808. 10.1113/jphysiol.2013.269977 24535444 PMC4001753

[B10] Goo S. Han J.-C. Nisbet L. A. LeGrice I. J. Taberner A. J. Loiselle D. S. (2014b). Dietary supplementation with either saturated or unsaturated fatty acids does not affect the mechanoenergetics of the isolated rat heart. Physiol. Rep. 2:e00272. 10.1002/phy2.272 24760525 PMC4002251

[B11] Goo S. Pham T. Han J.-C. Nielsen P. Taberner A. Hickey A.. (2013). Multiscale measurement of cardiac energetics. Clin. Exp. Pharmacol. Physiol. 40, 671–681. 10.1111/1440-1681.12139 23745944

[B12] Guild S.-J. McBryde F. D. Malpas S. C. (2015). Recording of intracranial pressure in conscious rats via telemetry. J. Appl. Physiol. 119, 576–581. 10.1152/japplphysiol.00165.2015 26159754

[B13] Hadi A. M. Mouchaers K. T. B. Schalij I. Grunberg K. Meijer G. A. Vonk-Noordegraaf A.. (2011). Rapid quantification of myocardial fibrosis: a new macro-based automated analysis. Cell. Oncol. 34, 343–354. 10.1007/s13402-011-0035-7 21538025 PMC3162624

[B14] Han J.-C. Barrett C. J. Taberner A. J. Loiselle D. S. (2015). Does reduced myocardial efficiency in systemic hypertensive-hypertrophy correlate with increased left-ventricular wall thickness? Hypertens. Res. 38, 530–538. 10.1038/hr.2015.37 25787044

[B15] Han J.-C. Goo S. Barrett C. J. Mellor K. M. Taberner A. J. Loiselle D. S. (2014a). The afterload-dependent peak efficiency of the isolated working rat heart is unaffected by streptozotocin-induced diabetes. Cardiovasc. Diabetol. 13:4. 10.1186/1475-2840-13-4 24387738 PMC3916799

[B16] Han J.-C. Taberner A. J. Kirton R. S. Nielsen P. M. F. Archer R. Kim N.. (2011). Radius-dependent decline of performance in isolated cardiac muscle does not reflect inadequacy of diffusive oxygen supply. Am. J. Physiol. Heart Circ. Physiol. 300, H1222–H1236. 10.1152/ajpheart.01157.2010 21217065

[B17] Han J.-C. Taberner A. J. Tran K. Goo S. Nickerson D. P. Nash M. P.. (2012). Comparison of the gibbs and suga formulations of cardiac energetics: the demise of “isoefficiency”. J. Appl. Physiol. 113, 996–1003. 10.1152/japplphysiol.00693.2011 22879535 PMC3487497

[B18] Han J.-C. Tran K. Johnston C. M. Nielsen P. M. F. Barrett C. J. Taberner A. J.. (2014b). Reduced mechanical efficiency in left-ventricular trabeculae of the spontaneously hypertensive rat. Physiol. Rep. 2:e12211. 10.14814/phy2.12211 25413328 PMC4255817

[B19] Han J.-C. Tran K. Nielsen P. M. F. Taberner A. J. Loiselle D. S. (2014c). Streptozotocin-induced diabetes prolongs twitch duration without affecting the efficiency of isolated ventricular trabeculae. Cardiovasc. Diabetol. 13:79. 10.1186/1475-2840-13-79 24731754 PMC4005834

[B20] Hardziyenka M. Campian M. E. Reesink H. J. Surie S. Bouma B. J. Groenink M.. (2011). Right ventricular failure following chronic pressure overload is associated with reduction in left ventricular mass: evidence for atrophic remodeling. J. Am. Coll. Cardiol. 57, 921–928. 10.1016/j.jacc.2010.08.648 21329838

[B21] Hardziyenka M. Campian M. E. Verkerk A. O. Surie S. van Ginneken A. C. G. Hakim S.. (2012). Electrophysiologic remodeling of the left ventricle in pressure overload-induced right ventricular failure. J. Am. Coll. Cardiol. 59, 2193–2202. 10.1016/j.jacc.2012.01.063 22676940

[B22] Hessel M. H. M. Steendijk P. den Adel B. Schutte C. I. van der Laarse A. (2006). Characterization of right ventricular function after monocrotaline-induced pulmonary hypertension in the intact rat. Am. J. Physiol. Heart Circ. Physiol. 291, H2424–H2430. 10.1152/ajpheart.00369.2006 16731643

[B23] Honda M. Yamada S. Goto Y. Ishikawa S. Yoshikane H. Ishinaga Y.. (1992). Biochemical and structural remodeling of collagen in the right ventricular hypertrophy induced by monocrotaline. Jpn. Circ. J. 56, 392–403. 10.1253/jcj.56.392 1533690

[B24] Hsia H. H. Haddad F. (2012). Pulmonary hypertension: a stage for ventricular interdependence? J. Am. Coll. Cardiol. 59, 2203–2205. 10.1016/j.jacc.2011.12.049. 22676941

[B25] Ishikawa S. Honda M. Yamada S. Goto Y. Morioka S. Ishinaga Y.. (1992). Different biventricular remodelling of myosin and collagen in pulmonary hypertension. Clin. Exp. Pharmacol. Physiol. 19, 723–732. 10.1111/j.1440-1681.1992.tb00410.x 1424302

[B26] Johnston C. M. Han J.-C. Ruddy B. P. Nielsen P. M. F. Taberner A. J. (2015). A high-resolution thermoelectric-module-based calorimeter for measuring the energetics of isolated ventricular trabeculae at body temperature. Am. J. Physiol. Heart Circ. Physiol. 309, H318–H324. 10.1152/ajpheart.00194.2015 26001412

[B27] Kögler H. Hartmann O. Leineweber K. Nguyen van P. Schott P. Brodde O.-E.. (2003). Mechanical load-dependent regulation of gene expression in monocrotaline-induced right ventricular hypertrophy in the rat. Circ. Res. 93, 230–237. 10.1161/01.RES.0000085042.89656.C7 12842921

[B28] Krayenbuehl H. P. Turina J. Hess O. (1978). Left ventricular function in chronic pulmonary hypertension. Am. J. Cardiol. 41, 1150–1158. 10.1016/0002-9149(78)90872-X 665524

[B29] Lamberts R. R. Vaessen R. J. Westerhof N. Stienen G. J. M. (2007). Right ventricular hypertrophy causes impairment of left ventricular diastolic function in the rat. Basic Res. Cardiol. 102, 19–27. 10.1007/s00395-006-0620-5 16944361

[B30] Loiselle D. S. Han J.-C. Mellor K. M. Pham T. Tran K. Goo S. (2014). Assessing the efficiency of the diabetic heart at subcellular, tissue and organ levels. J. Gen. Pract. 2:168 10.4172/2329-9126.1000168

[B31] Louie E. K. Rich S. Brundage B. H. (1986). Doppler echocardiography assessment of impaired left ventricular filling in patients with right ventricular pressure overload due to primary pulmonary hypertension. J. Am. Coll. Cardiol. 8, 1298–1306. 10.1016/S0735-1097(86)80300-X 3782636

[B32] Lourenço P. Roncon-Albuquerque R. Jr. Brás-Silva C. Faria B. Wieland J. Henriques-Coelho T.. (2006). Myocardial dysfunction and neurohumoral activation without remodeling in left ventricle of monocrotaline-induced pulmonary hypertensive rats. Am. J. Physiol. Heart Circ. Physiol. 291, H1587–H1594. 10.1152/ajpheart.01004.2005 16679394

[B33] Lurz P. Puranik R. Nordmeyer J. Muthurangu V. Hansen M. S. Schievano S.. (2009). Improvement in left ventricular filling properties after relief of right ventricle to pulmonary artery conduit obstruction: contribution of septal motion and interventricular mechanical delay. Eur. Heart J. 30, 2266–2274. 10.1093/eurheartj/ehp258 19561027

[B34] Manders E. Bogaard H.-J. Handoko M. L. van de Veerdonk M. C. Keogh A. Westerhof N.. (2014). Contractile dysfunction of left ventricular cardiomyocytes in patients with pulmonary arterial hypertension. J. Am. Coll. Cardiol. 64, 28–37. 10.1016/j.jacc.2014.04.031 24998125

[B35] Marcus J. T. Vonk Noordegraaf A. Roeleveld R. J. Postmus P. E. Heethaar R. M. Van Rossum A. C.. (2001). Impaired left ventricular filling due to right ventricular pressure overload in primary pulmonary hypertension: noninvasive monitoring using MRI. Chest J. 119, 1761–1765. 10.1378/chest.119.6.1761 11399703

[B36] Mauritz G.-J. Vonk-Noordegraaf A. Kind T. Surie S. Kloek J. J. Bresser P.. (2012). Pulmonary endarterectomy normalizes interventricular dyssynchrony and right ventricular systolic wall stress. J. Cardiovasc. Magn. Reson. 14, 1–9. 10.1186/1532-429X-14-5 22240072 PMC3305662

[B37] Menzel T. Wagner S. Kramm T. Mohr-Kahaly S. Mayer E. Braeuninger S.. (2000). Pathophysiology of impaired right and left ventricular function in chronic embolic pulmonary hypertension: changes after pulmonary thromboendarterectomy. Chest 118, 897–903. 10.1378/chest.118.4.897 11035654

[B38] Meyer M. (2014). Left ventricular atrophy in pulmonary arterial hypertension: a sinister dexter conundrum. J. Am. Coll. Cardiol. 64, 38–40. 10.1016/j.jacc.2014.04.027 24998126

[B39] Neely J. Liebermeister H. Battersby E. Morgan H. (1967). Effect of pressure development on oxygen consumption by isolated rat heart. Am. J. Physiol. 212, 804–814. 10.1152/ajplegacy.1967.212.4.804 6024443

[B40] Pham T. Tran K. Mellor K. M. Hickey A. Power A. Ward M.-L.. (2017). Does the intercept of the heat–stress relation provide an accurate estimate of cardiac activation heat? J. Physiol. 595, 4725–4733. 10.1113/JP274174 28455843 PMC5509849

[B41] Sanyal S. N. Wada T. Yamabe M. Anai H. Miyamoto S. Shimada T.. (2012). Cardiac autonomic nerve abnormalities in chronic heart failure are associated with presynaptic vagal nerve degeneration. Pathophysiology 19, 253–260. 10.1016/j.pathophys.2012.07.004 22921612

[B42] Stool E. W. Mullins C. B. Leshin S. J. Mitchell J. H. (1974). Dimensional changes of the left ventricle during acute pulmonary arterial hypertension in dogs. Am. J. Cardiol. 33, 868–875. 10.1016/0002-9149(74)90634-1 4829369

[B43] Taberner A. J. Han J.-C. Loiselle D. S. Nielsen P. M. F. (2011). An innovative work-loop calorimeter for *in vitro* measurement of the mechanics and energetics of working cardiac trabeculae J. Appl. Physiol. 111, 1798–1803. 10.1152/japplphysiol.00752.2011 21903883

[B44] Taberner A. J. Johnston C. M. Pham T. Han J.-C. Ruddy B. P. Loiselle D. S.. (2015). Measuring the mechanical efficiency of a working cardiac muscle sample at body temperature using a flow-through calorimeter, in 37th Annual International Conference of the IEEE Engineering in Medicine and Biology Society. Milan. 10.1109/EMBC.2015.7320240 26738140

[B45] Tran K. Han J. C. Crampin E. J. Taberner A. J. Loiselle D. S. (2017). Experimental and modelling evidence of shortening heat in cardiac muscle. J. Physiol. 595, 6313–6326. 10.1113/JP274680 28771742 PMC5621496

[B46] Wang Y. Jing L. Zhao X.-M. Han J.-J. Xia Z.-L. Qin S.-C.. (2011). Protective effects of hydrogen-rich saline on monocrotaline-induced pulmonary hypertension in a rat model. Respir. Res. 12, 26–26. 10.1186/1465-9921-12-26 21375753 PMC3065415

[B47] Wong Y. Y. Ruiter G. Lubberink M. Raijmakers P. G. Knaapen P. Marcus J. T.. (2011). Right ventricular failure in idiopathic pulmonary arterial hypertension is associated with inefficient myocardial oxygen utilization. Circ. Heart Fail. 4, 700–706. 10.1161/CIRCHEARTFAILURE.111.962381 21900188

